# A Case Report of a Malignant Peripheral Nerve Sheath Tumor of the Oral Cavity in Neurofibromatosis Type 1

**DOI:** 10.1155/2012/936735

**Published:** 2012-11-06

**Authors:** Özmen Öztürk, Alper Tutkun

**Affiliations:** ^1^Department of Otorhinolaryngology, School of Medicine, Istanbul Medipol University, Istanbul, Turkey; ^2^Kulak Burun Bogaz Anabilim Dalı, Istanbul Medipol Universitesi, Kosuyolu, Kadikoy, 34718 Istanbul, Turkey; ^3^Department of Otorhinolaryngology, Academic Hospital, Istanbul, Turkey

## Abstract

Patients with neurofibromatosis type 1 develop both benign and malignant tumors at an increased frequency. Most of the malignant peripheral nerve sheath tumors (MPNSTs) are considered as high-grade sarcomas originating from tissues of mesenchymal origin. It is generally accepted that MPNSTs occur in about 2% to 5% of neurofibromatosis patients. In this paper, we present a 16-year-old male patient with neurofibromatosis who developed MPNST of the retromolar area. The mass enlarged rapidly in a period of 6 weeks. The patient was treated surgically, and a tumor mass with a diameter of 7 × 6 × 4 cm was excised, but after 8 months a recurrence was observed at the same site. The sarcomatous change in a neurofibroma has an extremely poor prognosis, so patients with neurofibromatosis should be closely monitored for a possible malignancy. A rapid change in size of a preexisting neurofibroma, infiltration of the adjacent structures, intralesional hemorrhage, and pain indicate a possible malignant transformation to MPNST.

## 1. Introduction

Neurofibromatosis type 1 (NF1), an autosomal dominant neurocutaneous disorder, is commonly identified by the development of café-au-lait pigmentation of the skin, axillary freckling, optic nerve gliomas, Lisch nodules, distinctive bone lesions, and cutaneous or subcutaneous tumors called neurofibromas [1]. Patients with NF1 develop both benign and malignant tumors at an increased frequency [2].

 Neurofibromas are the most common benign tumors in NF1 [1, 2]. These tumors are composed of an admixture of Schwann cells, perineural fibroblasts, and mast cells infiltrating the peripheral nerves [1, 2]. Malignant peripheral nerve sheath tumors (MPNSTs) may develop within an associated neurofibroma [2, 3]. The malignant transformation of a neurofibroma has an extremely poor prognosis with prevalent recurrences and distant metastasis [2–4].

 In this paper, we present a 16-year-old male patient with NF1 who developed MPNST of the retromolar area. 

## 2. Case Report 

**Figure 1:**
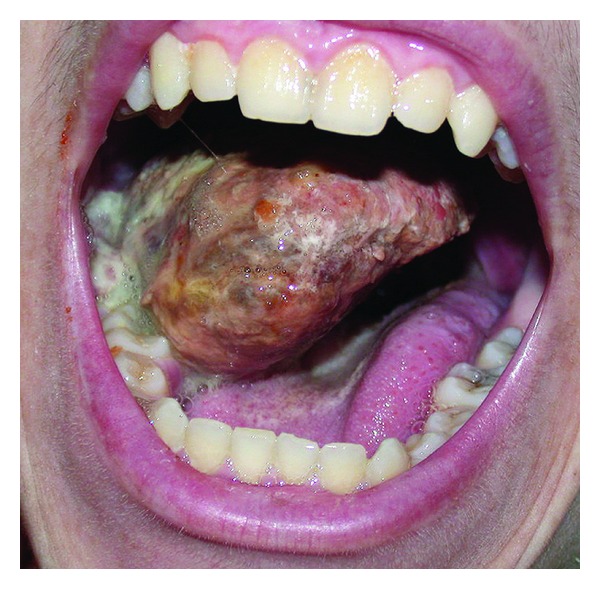
Oral examination showed a mass with nodular tissue causing severe dysphagia and respiratory distress.

A 16-year-old male patient with documented NF1 was referred with a rapidly enlarging mass in the mouth causing severe dysphagia, mandibular and temporomandibular pain, and respiratory difficulty (Figure 1). The patient first felt the presence of a nodular mass on the posterior part of his left buccal area with local numbness, but the mass enlarged progressively during a period of 6 weeks.

 The patient's clinical history started at the age of 3 years with the complaint of walking difficulty. At 4 years of age, a 3 × 2 cm mass was recognized on the left gluteal region. A general body scanning with imaging showed a preorbital and a pelvic mass in addition to the mentioned gluteal mass. At the age of 5 years, the patient had spinal laminectomy for the excision of a neurofibroma infiltrating S2 and S3 roots. The patient had multiple and recurrent neurofibroma excisions (4 times from the left gluteal region and once from the left lower abdominal region) in the following 2 years. At the age of 10, a Lisch nodule of the right eye was revealed. The patient had interferon-*α* therapy 3 times between the ages of 11 and 14. The family history of the patient was positive for NF1. His mother had documented NF1 with multiple neurofibromas and café-au-lait spots.

 His general physical examination showed a mass of the right gluteal region and thigh with an incision scar, multiple decubitus ulcers and necrotic areas present bilaterally on the gluteal regions and trochanters, a mass and an incision scar of the left lower quadrant of the abdomen, gynecomastia, multiple neurofibromas of the chest and trunk, multiple and diffuse café-au-lait spots of the axillary and gluteal areas and trunk (more than 10 spots determined to be larger than 1.5 cm), and scoliosis. The ophthalmologic examination revealed an optic glioma of the proptotic left eye and a Lisch nodule of the right eye.

**Figure 2:**
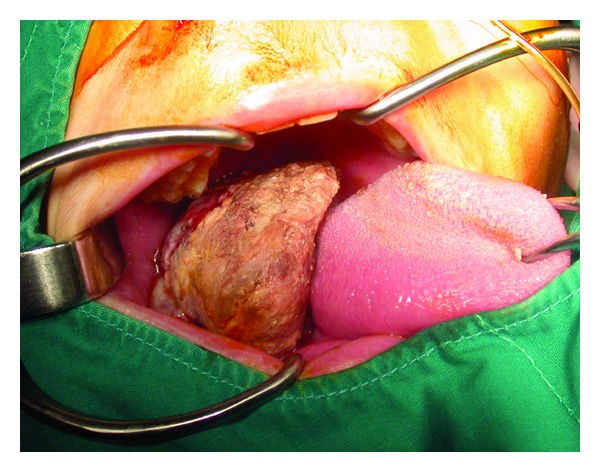
The preoperative examination showed a mass of the right retromolar area with a diameter of 7 × 6 × 4 cm extending deeply from the posterior ridge of the left lower alveolar process and gingivobuccal sulcus.

**Figure 3:**
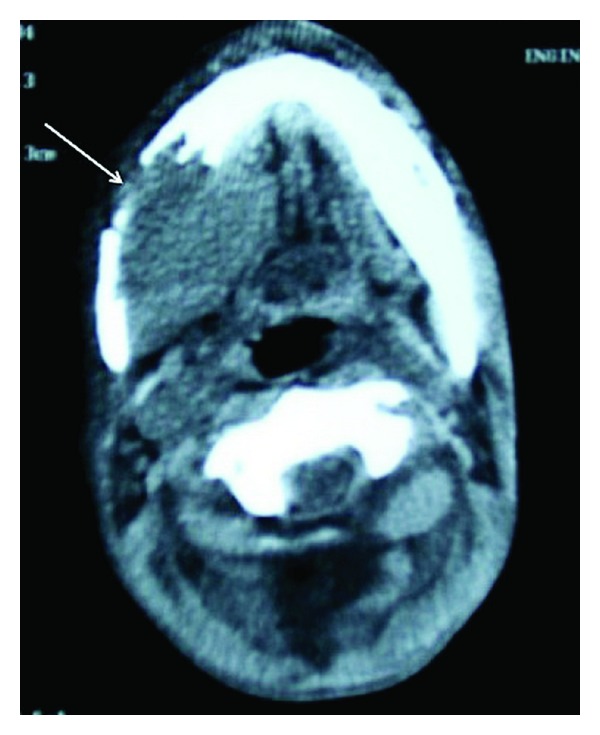
A CT scanning showed a mass with a diameter of 5 cm infiltrating the retromolar and posterior buccal areas with an erosion the mandibular alveolar ridge.

 The otolaryngologic examination showed a mass of the right retromolar area with a diameter of 6 × 5 × 3 cm extending deeply from the posterior ridge of the left lower alveolar process and gingivobuccal sulcus (Figure 2). Head and neck examination was not remarkable and no lymphadenopathies were palpated. A CT scanning showed a relatively homogeneous mass with a diameter of 5 cm infiltrating the retromolar and posterior buccal areas with an erosion the mandibular alveolar ridge (Figure 3). The tongue and the floor of mouth were found to be intact. A lymphadenopathy with a diameter of 12 mm was detected deep in the right jugulodigastric area.

**Figure 4:**
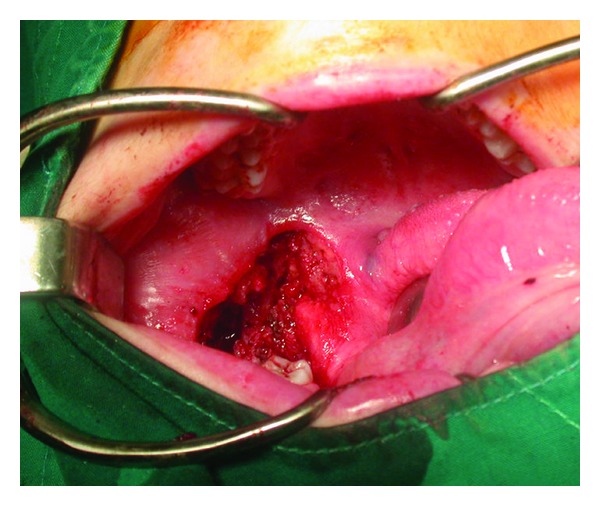
Postoperative appearance of the operative site.

 The mass infiltrating the left retromolar area and the posterior part of the lower alveolar process was excised with wide free margins (Figure 4). The first and second molar teeth (tooth numbers 18 and 19, according to universal system) were included with the specimen. The pathologic examination of the specimen with a diameter of 7 × 6 × 4 cm was reported as “MPNST,” with positive surgical margins at the inferior border of the tumor. The patient was followed up with no early complications related to the surgery.

 After 8 months, a mass with a diameter of 1 × 1 cm recurred in the same site. A cautious and conservative follow-up was planned, but the patient died in the intensive care unit 10 months after the operation due to sepsis following a severe infection of a large decubitus ulcer involving the sacral and gluteal regions. 

## 3. Discussion 

Oral manifestations are seen in approximately 72% to 92% of cases with NF1, usually presenting with an enlargement of the tongue and submucosal neurofibroma nodules [4]. The oral neurofibromas are usually diagnosed in teenagers, although all ages are susceptible [1]. They present as submucosal and painless soft masses that vary greatly in size, ranging from 2 cm to 8 cm. Jaw and mandible may be involved in 58% of the patients [5]. 

 A rapid change in size of a preexisting neurofibroma, compression or infiltration of the adjacent structures, hemorrhage, and unremitting pain indicate a possible malignant degeneration to MPNST [2]. Most MPNSTs are considered high-grade sarcomas originating from tissues of mesenchymal origin. Histopathology reveals malignant tissue composed of spindle cells arranges in cellular fascicles and a mixture of poorly defined cellular and cystic components expressing vimentin and S-100 [2, 3]. Its features reveal a fusiform or globoid mass with necrosis, pseudocystic change, or hemorrhage [3]. 

MPNSTs comprise almost 10% to 12% of all soft tissue sarcomas, which are primarily associated with NF1 [3]. It is generally accepted that MPNSTs occur in about 2–5% of NF1 patients compared with an incidence of 0.001% in the general population [2]. Most of the patients present in the second and third decades of life, but when NF1 is present MPNST tends to occur in younger patients [2]. Findings on imaging studies define location and extention of the disease and reveal metastasis. Fine needle aspiration, Tru-Cut needle biopsy, and incisional and excisional biopsies are used for diagnosis. 

 Treatment is predominantly surgical [2–4]. The goal of the operation is to achieve complete surgical excision of the tumor with negative margins. This offers the best outcome with respect to both local recurrence and distant metastases. Radiation therapy can be employed in preoperative, intraoperative, and postoperative settings [2]. Treatment with adjuvant radiotherapy has yielded a statistically significant reduction in the rates of local disease recurrence [2].

 The neurofibrosarcomatous change in a neurofibroma brings about an extremely poor prognosis, and recurrences and distant metastasis are common [2–4]. Studies have shown the average 5-year survival rate for these patients ranges from 16% to 52% [6]. NF1 patients should be closely monitored for malignant transformation so early recognition and surgical intervention can be initiated. Defining the characteristics of MPNST on a molecular level and genetic counseling might allow a precise screening test and an earlier disease detection.
